# Health situation of migrant and minority nurses: A systematic review

**DOI:** 10.1371/journal.pone.0179183

**Published:** 2017-06-26

**Authors:** Benjamin Schilgen, Albert Nienhaus, Oriana Handtke, Holger Schulz, Mike Mösko

**Affiliations:** 1 Department of Medical Psychology, University Medical Center Hamburg-Eppendorf, Hamburg, Germany; 2 Competence Centre for Epidemiology and Health Service Research in Nursing, Institute for Health Service Research in Dermatology and Nursing, University Medical Centre Hamburg-Eppendorf, Hamburg, Germany; 3 Department of Occupational Health Research, German Social Accident Insurance Institution for the Health and Welfare Services, Hamburg, Germany; Western Sydney University, AUSTRALIA

## Abstract

**Introduction:**

Globally, life expectancy together with multimorbidity and chronic diseases are increasing. This leads to a growing demand for care and hence for healthcare personnel and nurses. To meet this demand, healthcare workers from abroad are increasingly hired. The nurses’ workplace in general is characterized by physically and psychologically demanding tasks, while that of migrant and minority nurses is additionally characterized by discriminatory practices. The present knowledge about the health of migrant and minority nurses and the terminology in this context are diverse. Thus, the purpose of this review is to systematically identify and synthesize international publications that explicitly focus on migrant nurses’ health.

**Materials and methods:**

A systematic review of relevant studies was undertaken using the databases Medline, PsycINFO, CINAHL and Web of Science. The screening process was conducted in several phases. This review was conducted in accordance with the Preferred Reporting Items for Systematic Reviews and Meta-Analyses (PRISMA) guidelines while the methodological quality assessment of the included papers was performed with the Mixed Method Appraisal Tool (MMAT).

**Results:**

Out of 11,599 citations initially obtained, 14 empirical studies were included in the final synthesis. The methodological quality of the empirical studies and reviews was diverse. The majority of the studies were conducted in the US and the nurses under study migrated from countries like the Philippines, India, Europe, and Africa. Among migrant nurses of different origins, there are differences in their physiological responses to stress. Migrant nurses and native nurses differ in reporting work-related injuries.

**Discussion:**

Migrant and minority nurses are at high risk of work-related injuries and discrimination than native or majority nurses. However, mixed results were obtained, namely that the reported health of migrant nurses either improves over time or it decreases. This review revealed that discrimination is the leading cause of impaired health amongst migrant and minority nurses.

## Introduction

According to recent estimates of the International Labour Organization, there are 150.3 million migrant workers worldwide, nearly one fourth of which are located in North America as well as Northern and Western Europe. More than half of the overall number of migrant workers are engaged in the service sector, followed by the industrial and agricultural sector. Nearly 10% are employed as domestic workers, such as live-in caregivers or housemaids [1]. In the context of international mobility, the number the number of migrant nurses and doctors immigrating to OECD countries from countries with severe shortages in the health workforce (e.g. Angola, Ethiopia, Haiti, Pakistan, Cambodia or Indonesia) increased by 80% between 2000 and 2010 [2, 3]. The working situation of migrant and minority workers is characterized by temporary and part-time jobs as well as informal employment [4]. In addition migrant and minority workers are overrepresented in less skilled occupations [4, 5]. Migrant and minority workers are also at high risk of discrimination in hiring and layoffs and also face exploitation in terms of unequal payment, longer working hours at the workplace and restricted access to workplace benefits [5–8]. The risk of fatal and non-fatal injuries is higher for migrant and minority workers than for native workers [9], even if the occupational category is controlled [10]. The findings about the mental health of migrant workers show contradictory results. For example, while migrant workers in China show better mental health, migrant workers in Sweden reported increased levels of mental distress [11, 12].

Due to a global increase in life expectancy together with multimorbidity and chronic diseases the demand for care, and hence for nurses and other healthcare personnel in old age care is growing steadily [13–15]. The healthcare workforce itself is also not only ageing, but the growing demand is also confronted with declining supply [5, 16]. In addition to the strategy to educate and train domestic healthcare professionals, developed countries try to attract healthcare workers from abroad to meet the growing demand [6, 17].

The number of migrant nurses and doctors has grown by 60% within the Organisation for Economic Co-operation and Development (OECD) since 2004 [1]. New Zealand, Switzerland, Australia and Luxembourg register the highest proportion of foreign-born nurses within the OECD with more than 30%. In the United States and in Germany 14% of nurses are not native. India and China send the largest proportion of doctors to OECD countries. Among the OECD European countries, Germany and the United Kingdom account for the largest number of emigrating doctors and nurses. [1, 18]. With reference to foreign-born nurses within the OECD, the Philippines and India accounted for the highest shares [1, 18].

The migration process can be associated with specific stressors causing adverse effects on the migrant’s health. On the one hand the “exhausted migrant effect” [19], or the acculturation stress hypothesis [20], argues that the lower state of migrants' health compared to non-migrants is caused by impaired living and working conditions, separation from family, loss of social structures and support networks or social exclusion [21]. Studies showed that the level of acculturation has influence on the individual’s social behaviour—e.g. the lower the level is, the higher is the risk for stress in a relationship or conflicts with family members and friends [22]. The effect of acculturation on health outcomes has been shown by studies, in which traditionally-oriented migrants are more likely to have type 2 diabetes than their assimilated counterparts [22].

On the other hand, the “healthy-migrant hypothesis” or “selective migration theory” states that migrants are in better health than their native counterparts. This effect can be explained by the better health and wealth of those people who decide to migrate. These migrants are more willing to take the challenge that inheres in the process of migration in terms of unfamiliar culture, behaviours or language. While the sending country loses healthy people, the receiving country’s population benefits from those in terms of an influx of younger and more healthy people [23].

There is evidence that the mental health develops quite differently among migrants. According to the “U-Curve theory” [24] migrants feel euphoric and fascinated in the first year of their migration. This is followed by a period of disillusionment and frustration due to the challenge of coping with the new culture in daily life. Adapting gradually to these challenges makes migrants finally able to master their life in the new environment and culture. However, as studies show, migrants also suffer from anxiety and depression the longer they live in their host country [25].

Migrants report language barriers, lack of knowledge of cultural diversity, lacking trust and mutual respect as well as lack of knowledge. These are a few among several obstacles that can impede a working relationship between migrants and autochthonous people. Becoming culturally competent is an important step for healthcare providers to manage the challenges arising from a growing diversity [26]. It has been shown that cultural competence programs in ethnic minority patient-centered health care do not automatically lead to better practice and so to improved health-related outcomes on the side of patients. Nonetheless, these programs led to an increased knowledge and awareness how to deal with culturally diverse patients [27].

Migrant health is impeded by additional stressors not shared by their native colleagues [28]. Beyond the excessive job stress, burnout and job dissatisfaction that migrant and native employees share, foreign doctors experience discrimination, unknown norms and behavioural patterns and limited setting-specific knowledge at their workplace [29, 30]. A Finnish study revealed that migrant doctors perceive more work-related distress and are at twice the risk of burnout compared to their native colleagues [31]. Foreign-born family therapists are confronted with stereotypical behaviour and they feel they are perceived as less sympathetic by their clients [32].

The workplace of nurses is characterized by physically and psychologically demanding tasks and biological hazards, like acquiring hand dermatitis due to continuous use of gloves [33, 34]. They are at a high risk of musculoskeletal pain that can be compounded by work-related psychosocial factors. They are facing several stressors like high demands/low control, effort-reward imbalance and low social support, which are associated with pain in the upper and lower extremities [35]. Back pain is the leading cause of limited physical performance at work, as well as for nurses’ absenteeism [36]. Occupational stress among nurses presents a considerable risk of sleep disorder [37]. Nurses report poor working environments that lead to job dissatisfaction and burnout [38].

Migrant and minority nurses additionally report discrimination and racism at work [39–41] in terms of a lack of employment opportunities [42], poor career progression [43] or poor learning environment [44, 45].

They experience racial discrimination in contact with patients and their families, but also with doctors, management and nursing colleagues [46]. Discrimination and racial harassment have a significant negative impact on job satisfaction [47]. This effect may diminish over time, as in one study Filipino nurses feel more satisfied with their job due to prolonged acculturation in the US [48]. Discrimination at workplace is also associated with unhealthy behaviours such as smoking and negative job outcomes like self-reported and medically-certified sickness absence. Racial/ ethnic discrimination may lead to poor mental health, psychological distress as well as anxiety and depression as Okechukwu et al. (2014) explained [49].

In summary, it can be stated that there is a broad variety of evidence that focuses on migrant workers facing specific work-related health risks. Workplace retention is positively correlated with the health of nurses, so providing them with a healthy workplace increases the retention rate [50–52].

However, to date no systematic review that focuses on the health of migrant and minority nurses has been undertaken. The purpose of this review is to systematically identify and synthesize international publications to gain understanding of work-related health risks and their impact on migrant and minority nurses' health.

## Materials and methods

The research question underlying the review was “What do we know about the health of migrant and minority nurses?”. The review was conducted in accordance with the Preferred Reporting Items for Systematic Reviews and Meta-Analyses (PRISMA) guidelines [53].

### Search strategy

The search strategy included a systematic search of electronic databases. Additionally, reference lists of included studies were manually searched for additional citations. To systematically identify terms that map the broad spectrum of the research question, the PICO criteria were adapted [54] and focused on the criteria population (‘migrant nurses’) and outcome (‘health’). Terms in the context of migrant nurses are used interchangeably and inconsistently across the healthcare literature and so a distinct terminology in relation to migrant nurses is missing. Therefore, the population criterion was first divided into the content categories ‘nurse’ and ‘migration and minority’ and the outcome ‘health’ into ‘health in general’ and ‘determinants of health’. These categories were then each equipped with corresponding search terms. The category ‘nurse’ was determined by terms like nurse, nurse aide or home nurse, while terms like multicultural, overseas qualified, minority or ethnic, foreign or migrant workforce formed the category ‘migration’. Within the category ‘health in general’ terms like health, mental health and disease were used. The category ‘determinants of health’ consists of more specific health-related terms that in turn were subdivided into physical (e.g. musculoskeletal pain, smoking, sleep) and psychosocial (e.g. resilience, stress, job satisfaction) determinants of health.

The search terms were identified via an analysis of the Medical Subject Headings (MeSH) and text words of key articles identified a priori. The search terms were then discussed among the authors of the review and then compiled into a search string for Medline. The search string was iteratively adjusted to the other search databases PsycINFO, CINAHL and Web of Science.

The electronic database searches were conducted between April and May 2016. To reach the whole scope of published literature within each of the databases, a date restriction was not set. Titles and abstracts published in German or English were included. Any primary or secondary research studies that are conducted in a qualitative or quantitative manner were included in the full-text screening. While books, as well as commentaries, policy statements or letters to the editor were excluded, doctoral theses were screened for eligibility. The references were managed with EndNote X7 to assure a lossless processing of the data reduction. The exhaustive search string can be found in S1 Appendix.

### Eligibility criteria and assessment

The selection process was divided into two phases that were subdivided into stages. The first phase was characterized by being as comprehensive as possible. Thus, following the broad and diverse research question, titles and abstracts were included once they focused on ‘migration’ and ‘nurses’ and ‘health’. The screening process of titles and abstracts was split, so that the main author and a further member of the research group each screened 250 randomly chosen articles and reached an interrater reliability of ĸ = 0.9. Differences were analysed between both raters. One quarter of the retrieved references were then screened by the second rater, while three quarters were screened by the main author. Regular meetings between both raters were held throughout the screening process to ensure a high level of consensus and to discuss any uncertainties [55].

Selection phase two (full-text screening) was subdivided into two stages. A set of specific eligibility criteria was developed and iteratively adapted for this selection phase [56]. The eligibility criteria set was divided into the categories design, population and content [57]. Within the first stage of full-text screening any empirical studies or reviews with a population size greater than 1, but no commentaries or policy statements or books or letters to the editor were considered under the category ‘design’. The category ‘population’ screened for migrant and minority nurses as professional caregivers. Family caregivers as well as home health aides were excluded. ‘Migrants’ were identified as those who themselves or whose parents exhibit a foreign birthplace or those who speak a foreign native tongue or have a foreign nationality or those who are referred to as ‘migrants’ or ‘immigrants’ or ‘foreign’ [58]. Minority nurses were identified as those who are referred to as ‘minority’ or ‘ethnic minority’ or ‘racial minority’ To meet the ‘content’ criterion, relevant studies had to examine the state of health (mental, psychosocial, and physical) or health-related factors that might influence the individual’s health positively or negatively (e.g. team support, high load of work, satisfaction). Migrant nurses had to be clearly identifiable within the article and descriptions of health had to be distinctly attributed to this group.

**Fig 1:**
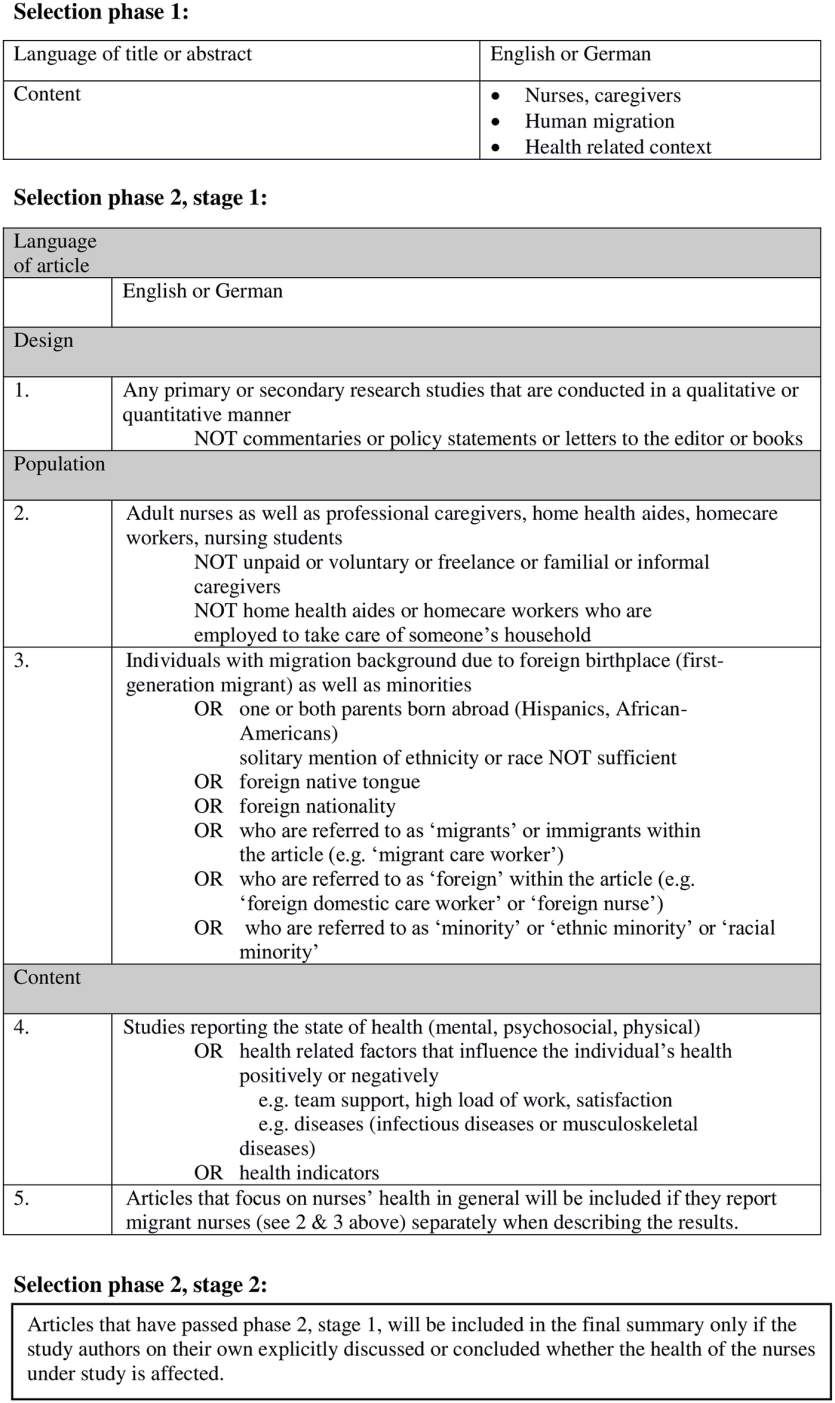
Overall inclusion and exclusion criteria for selection phases 1 and 2.

After having completed the full-text screening, the inclusion criteria were adapted, so that only articles were finally included in which the study authors on their own explicitly discussed or concluded whether the health of the nurses under study is affected. This adaption has been made to assure that this review explicitly focus on the health of migrant and minority nurses. (Fig 1). The list with excluded articles and the reasons for exclusion can be found in S2 Appendix. Within the second selection phase both raters reached an interrater reliability of ĸ = 0.96 after an initial screening of 50 independently chosen articles. To ensure a high level of consensus in the full-text screening, both raters held regular meetings to discuss possible differences [55]. All full-text articles were screened by both raters independently.

### Data extraction and quality assessment

The data extraction was performed by the main author and cross-checked by a further member of the research group. The methodological quality assessment of the included papers was independently performed and based on the Mixed Method Appraisal Tool (MMAT).

Both raters reached a good interrater reliability of ĸ = 0.8 for the assessment with the MMAT [59]. Regular meetings between both raters were held throughout the screening process to ensure a high level of consensus and to discuss any uncertainties [55].

Pluye et al. (2011) suggest scoring the risk of bias of quantitative, qualitative as well as mixed-methods studies with their MMAT tool [60].

### Data analysis and description

Due to the broad and diverse research question the results of this review were synthesized in a narrative and descriptive manner [56]. Study characteristics were summarized descriptively. The qualitative study findings were collated by applying a thematic analysis, whereas the quantitative study results were synthesised descriptively.

## Results

The review findings are presented in four ways. First the flow of the study selection is described. Then the study characteristics are illustrated. Thirdly the quality of these studies is highlighted and finally the studies are organized according to the themes that emerged from the thematic analysis.

### Study selection

**Fig 2:**
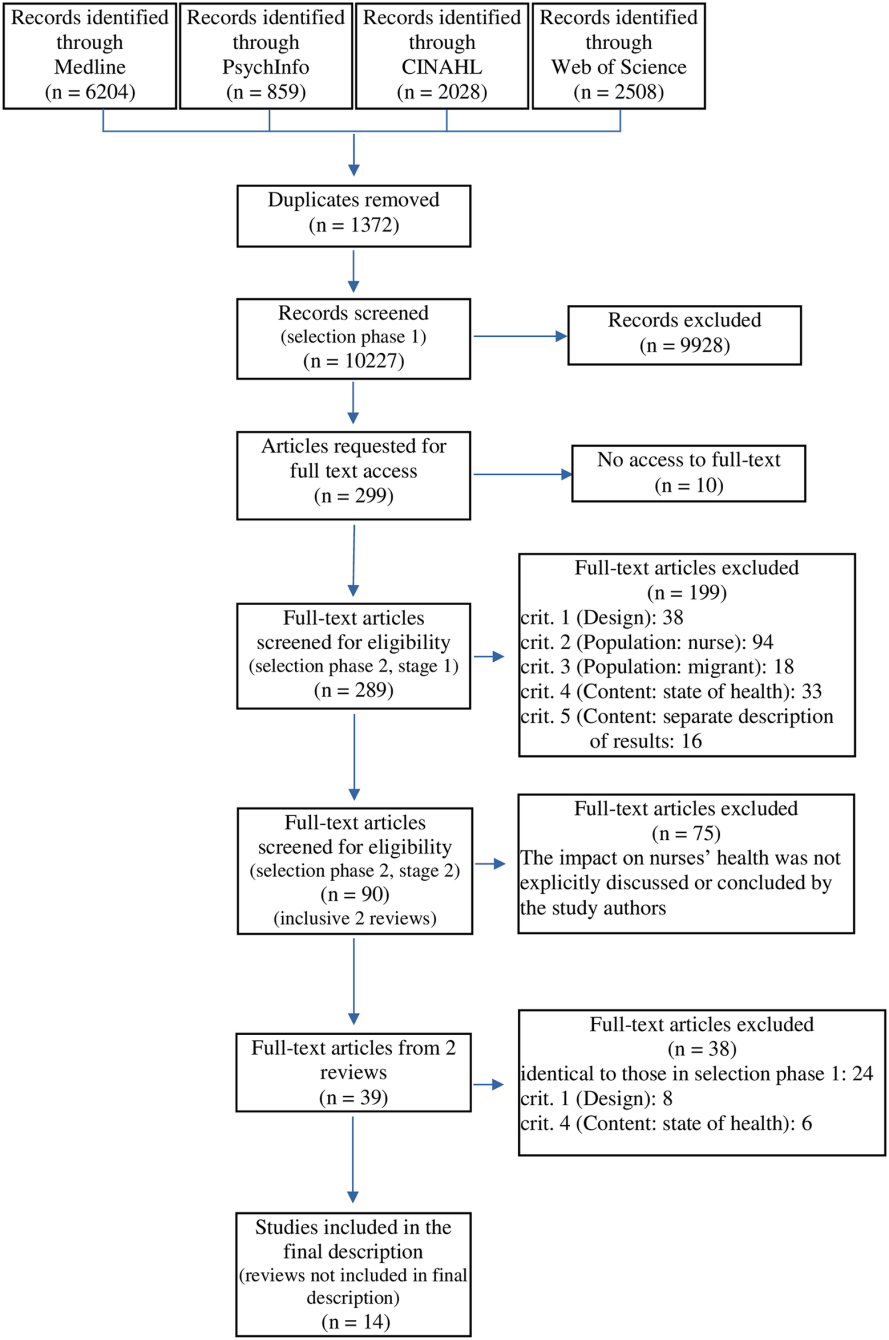
Flowchart of literature search. Source: Adapted from PRISMA flow diagram. crit. = criterion.

The databases search resulted in 11,599 citations. After having removed duplicates, 10,227 references were scanned according to title and abstract for inclusion. Of these, 299 met the eligibility criteria in the first selection phase. Ten articles could not be retrieved in full text despite elaborate efforts. In phase two 289 articles were read in full text and 90 met the inclusion criteria (Fig 2). 75 articles were excluded since the impact on nurses’ health was not explicitly discussed or concluded by the study authors. Of the remaining 15 articles, two were literature reviews containing 39 articles in total; those were scanned for eligibility and 24 articles were excluded since they were identical to those, that have been removed in selection phase one. The two literature reviews were not included in the final synthesis. Of the remaining 15 articles deriving from the two reviews, only one study met the inclusion criteria. Thus, the final data set comprised 14 articles with one study deriving from the two reviews. [40, 41, 61–72].

### Study characteristics

All articles were published in English between 1997 and 2013 and issued in journals, while one study was conducted in the context of a doctoral dissertation [64]. The majority of the studies were conducted in the US, but also in Canada, Denmark, England, Israel and Yemen. Six studies were carried out in hospitals, two in colleges; one was implemented across three sites (NHS trusts, participants’ home and restaurant), two in nursing homes and finally one study was administered via email. The target group of the studies were various nursing and allied professions such as professional nurses, nurse’s aides or nursing students. In most of the empirical studies the nurses were mainly female and their average age ranged from 21 to 51 years. The nurses under study migrated from Europe and other countries of origin like Africa, the Philippines, India, the former Soviet Union, Israel, or Mexico.

Out of 14 empirical studies eleven were cross sectional and two were longitudinal studies. Twelve of the studies employed quantitative and one qualitative methods, while two used a mixed-method design. Convenience and purposive sampling methods were most frequently applied to recruit the study participants, while Beechnior et Fitzpatrick (2008), Pittman et al. (2014) and Tak et al. (2010) recruited their study sample randomly [41, 71, 72].

Within the studies diverse health-related outcomes were examined. While six studies focussed on somatic outcomes [61–63, 65, 70, 71], eight articles addressed psychosocial outcomes [41, 64, 66–69, 72, 73]. Physiological responses of nurses to anxiety, anger and happiness [61–63] like elevated blood pressure were observed but also physical reactions like back pain [65]. Simpson and Severson (2000) and Tak et al. (2010) examined injury rates among nurses [70, 71]. As direct health-related psychosocial outcomes perceived stress at work, job strain and levels of depression [66, 68] and distress [72] were identified. The level of job satisfaction and autonomy [73] as an indirect health-related psychosocial outcome was assessed by Hayne et al. (2009). So did the studies of Hogh, Carneiro et al. (2011), Likupe and Archibong (2013) and Pittman, Davis et al. (2014) by focusing on experiences of discrimination, racism and bullying [41, 67, 69], while Diggs identified different coping styles of nursing students facing stress at work and university [64].

Diverse instruments were applied in the studies. Besides the job content questionnaire [66], the Nursing Work Index [73], the Occupation Stress Inventory [73], the Demands of immigration (D.I.) scale [72], the Ways of Coping Checklist [64], and the Beck Depression Inventory [61, 63, 67], self-developed questionnaires were used for data collection [41, 62, 65, 67, 69–71].

Characteristics of all articles are detailed in Tables 1–4. A more detailed overview of the characteristics (e.g. study objectives, participants’ inclusion criteria, statistical and outcome measures, descriptive results) of all studies can be accessed in S3 Appendix.

**Table 1:** Study characteristics of the included empirical studies: *Cross-sectional quantitative*.

reference	title	study setting		recruitment	design and method	sample					findings
		location	country	strategy	instrument	strategy	total n	origin/race/ethnicity (n)	age	professional status (n)	
[62]	Comparison of factors affecting daily variation of blood pressure in Filipino-American and Caucasian nurses in Hawaii	Hilo Medical Centre, Life Care Centre of Hilo	USA	on-site recruitment (workplace)	ABP monitor, mood scale	non-probability: convenience sample	60	Filipino-Americans (38)	33.8 ± 6.0	professional nurses, nurse’s aides	Filipino Americans had a slightly greater increase in systolic blood pressure at work relative to home than Caucasians. Filipino Americans reported more frequently negative moods at home as well as at work
								Caucasians (22)	38.1 ± 6.8		
[63]	Job strain and physiological stress responses in nurses and nurse’s aides: predictors of daily blood pressure variability	Hilo Medical Centre, Life Care Centre of Hilo	USA	on-site recruitment (workplace)	job content questionnaire, ABP monitor, anthropometric measurements, urine sample	non-probability: convenience sample	59	Filipino-Americans (36)	33.7 ± 6.1	professional nurses, nurse’s aides	Euro-American reported more likely job strain than Filipino-Americans. Euro-American showed a significantly higher mean score on the psychological demand subscale of the JCQ (P < 0.05)
								Euro-Americans (23)	37.9 ± 6.6		
[61]	Physiological stress responses in Filipino-American immigrant nurses: the effects of residence time, life-style, and job strain	Hilo Medical Centre, Hilo Life Care Centre	USA	subsample of Brown, James, et al. 1998	anthropometric measurements, ABP monitor, urine sample, self-designed questionnaire, job content questionnaire (Karasek et al. 1998)	not reported since subgroup	31	Filipinos	35.0 ± 6.0	nurse’s aides (20)	Longer residence in the United States led to elevated norepinephrine levels among nurses from the Philippines in the work and home settings, higher diastolic blood pressure during sleep, and lower dips in blood pressure during sleep.
									34.4 ± 5.2	professional nurses (11)	
[65]	Low back pain among female nurses in Yemen	4 major hospitals in Sana’a City	Yemen	study hospitals’ payroll lists used as a sampling frame	structured pre-coded questionnaire	random representative sample	687	Yemeni (332)	29.4 ± 6.6	professional nurses	Prevalence of low back pain was persistently higher among Yemeni nurses. The prevalence was significantly lower in Indian nurses compared to other nurses (P < 0.001). Although sharing the same working conditions, Indian nurses were less likely to report low back pain.
								Indian (346)			
								others (9)			
[72]	Demands of immigration among nurses from Canada and the Philippines	State of Hawaii	USA	not reported	Demands of immigration (D.I.) scale (Aroian et al. 1998)	random sample	73	Canadians (C)	20–30: 25% (C); 0% (P)	Registered nurses	Canadian nurses reported significantly higher total distress due to the demands of immigration than did Filipino nurses working in the U.S.
									31–40: 13% (C); 21% (P)		
								Philippines (P)	41–50: 37% (C); 45% (P)		
									>50: 25% (C); 34% (P)		
[68]	Racial disparities in job strain among American and immigrant long-term care workers	4 nursing homes in Massachusetts	USA	not reported	job Content Questionnaire (Karasek et al. 1998)	non-probability: purposive sample	237	Non-Hispanic Black (127) [Caribbean (72), Black (51)]	39.4 ± 10.4	registered nurses	Black certified nursing assistants reported more often job strain and low control. There is no difference between Black and White Registered nurses. Black workers earned less and worked more relative to White workers of the same occupation.
								Non-Hispanic White (110) [African (6)]	42.6 ± 12.2		
[41]	Perceptions of employment-based discrimination among newly arrived foreign-educated nurses	questionnaires administered online	USA	email	self-developed questionnaire	random sample	502	Filipinos (182)	Not reported	registered nurses	Nurses, that migrated from low income countries and those recruited by staffing agencies were at higher risk to receive unjust treatment compared with their U.S. counterparts in terms of wages, orientation at workplace and discrimination.
								other low-income countries (161)			
								Canada (138)			
								other high-income countries (21)			
[70]	Risk of injury in African American hospital workers	metropolitan hospital	USA	N/A	Occupational Health Service (OHS) medical records	Complete survey	2247	African-American (1203, injured:111)	<30: 369;	Housekeeping aide, Nurse’s aide, Radiology technologist, Staff nurse, Transportation aide, Unit clerk	African Americans were at higher risk of suffering work-related injuries than Whites.
									31–40: 664;		
									41–50: 699;		
								White (1044, injured:44)	51–60: 344;		
									>60: 171		
[71]	Racial and ethnic disparities in work-related injuries and socio-economic resources among nursing assistants employed in US nursing homes	Nursing homes in United States of America	USA	representative sample	pilot tested self-developed questionnaire with input from National Institute for Occupational Safety and Health; Occupational Safety and Health Administration, and Centers for Medicare & Medicaid Services)	random sample	2880	Non-Hispanic White (1351, 46.9%)	18–24: 17.0%;	certified nursing assistants	Among all racial and ethnic groups, Non-Hispanic white Nursing Assistants were more likely to report work-related injuries. Non-Hispanic black NAs were more likely to have a low household income, no health insurance and no pay raises.
								Non-Hispanic Black (1066, 37%)	25–34: 23.5%;		
								Hispanic (272, 9.4%)	35–44: 24.6%;		
								Other (191, 6.6%)	45–54: 22.6%;		
									>55: 12.2%		

**Table 2:** Characteristics of the included empirical studies: *Longitudinal quantitative*.

reference	title	study setting		recruitment	design and method	sample					findings
		location	country	strategy	instrument	strategy	total n	origin/race/ethnicity (n)	age	professional status (n)	
[66]	Stages of acculturation as reflected by depression reduction in immigrant nursing students	not reported	not reported	not reported	self-developed questionnaire	non-probability: convenience sample	75	Former Soviet Union	27 ± 8.1	registered nurses (26)	Mild levels of depression among Immigrant nursing students descended after 6 months to normal level. Immigrants reported an increasing social support by non-family members.
									21 ± 3.5	nursing students (16)	
					Beck Depression Inventory			Israel	24 ±5.2	nursing students (33)	
[67]	Are immigrants in the nursing industry at increased risk of bullying at work? A one-year follow-up study	healthcare colleges	Denmark	on-site recruitment (healthcare college)	self-developed questionnaire	Complete survey	T1: 5635	Danish (5040)	32.6	healthcare helper (HCH) students (elderly care)	Non-Western immigrant students were at higher risk of being bullied in college and trainee periods than Western immigrants. Both immigrant groups were at higher risk of being bullied by clients or residents than Danish nursing students.
								Non-Western immigrants (457)	33.6		
								Western immigrants (138)	38.1		
							T2: 3109	Danish (2831)	35.2	healthcare assistant (HCA) students (elderly and hospital care)	
								Non-Western immigrants (73)	37.3		
								Western immigrants (205)	39.2		

**Table 3:** Characteristics of the included empirical studies: *Cross-sectional qualitative*.

reference	title	study setting		recruitment	design and method	sample					findings
		location	country	strategy	instrument	strategy	total n	origin/race/ethnicity (n)	age	professional status (n)	
[69]	Black African Nurses’ Experiences of Equality, Racism, and Discrimination in the National Health Service	four NHS trusts in north-eastern England, participants’ homes, restaurants	England	advertisements in various wards to invite nurses for participation	self-developed interview guide	non-probability: purposive sample	30	Malawi, Kenya, Ghana, Nigeria, South Africa, Zambia, Zimbabwe, Cameroon	35 (25 to 48 yrs)	nurses (30)	Black African nurses experienced racism, discrimination and lack of opportunity in their workplace. These experiences caused Black African nurses considerable distress and confusion that may affect their health, levels of confidence and their self-esteem

**Table 4:** Characteristics of the included empirical studies: *Cross-sectional mixed methods*.

reference	title	study setting		recruitment	design and method	sample					findings
		location	country	strategy	instrument	strategy	total n	origin/race/ethnicity (n)	age	professional status (n)	
[64]	Coping and its relation to retention among male minority nursing students in an associate degree nursing programme in a South Texas community college: An explanatory sequential mixed methods inquiry	community college	USA	on-site recruitment (community college)	modified version of the Ways of Coping Checklist (WCC), (Folkman et al., 1986)	non-probability: purposive sample	39	Hispanic (Mexican) (28, 71.8%)	31	nursing students	Minority nursing students mostly employed planful problem-solving as a coping skill while escape-avoidance was the skill used the least. Challenges within the study progress motivated the students to finish their study program.
								Other Hispanic (1, 2.6%)			
								African-American (4, 10.3%)			
								Asian-American (6, 15.4%)			
					focus groups	non-probability: purposive sample (sampling of the quantitative cohort)	4	Hispanic (Mexican) (2)	not reported		
								Other Hispanic (1)			
								African-American (1)			
[73]	Filipino nurses in the United States: recruitment, retention, occupational stress, and job satisfaction	550-bed community hospital located in the United States	USA	participants were invited via the hospital e-mail system	self-developed interview guide	non-probability: purposive sample	3	Not reported	Not reported	Hospital President, Chief Nursing Officer, Staff Education Coordinator	Nurses felt supported to act in their patients’ best interest and to make independent judgments. Practicing professional nursing and having a a positive relationship with the medical staff also led to an overall positive job satisfaction.
					Nursing Work Index Revised Edition (Aiken & Patrician, 2000), Occupation Stress Inventory-Revised Edition (Osipow, 1998)		15	Filipinos	35.4 (30 to 51 yrs)	professional nurses	

### Quality assessment

The detailed methodological quality rating results of the empirical studies are displayed in Tables 5–8. Five studies met all criteria according to MMAT [65, 67, 69, 71, 72], three studies reached 3 out of 4 stars [41, 68, 70] and the remaining six met one or two criteria [61–64, 66, 73]. Among the quantitative descriptive studies, only Ghilan et al. (2013) and Tak et al. (2010) applied an adequate sampling strategy, provided a representative sample, used appropriate measurements and reached an acceptable response rate [65, 71]. The longitudinal study from Hogh et al. (2011) received robust rating results, since the study participants were recruited in a way that minimized selection bias and the measurements were appropriately designed [67]. Differences between groups and their possible impact on the results were considered and an acceptable response rate at follow-up was achieved. The one sole qualitative study by Likupe and Archibong (2013) reached a strong quality score. The study authors chose adequate sources of qualitative data as well as a corresponding data analysis strategy that matches the research objectives. The extent to which the study findings relate to the study context is precisely outlined, as is the authors’ influence on the study findings [69].

**Table 5:** Methodological quality of the included empirical studies (quantitative descriptive).

reference	Quantitative descriptive				Total Points	Score
	Sampling strategy relevant to objectives	Sample representativeness	Measurements appropriate	Acceptable response rate		
[41]	1	1	1	0	3/4	0.75 (***)
[65]	1	1	1	1	4/4	1.0 (****)
[68]	0	1	1	1	3/4	0.75 (***)
[71]	1	1	1	1	4/4	1.0 (****)

Score (MMAT):

*** (75%) = three out of four criteria met;

**** (100%) = all four criteria met

**Table 6:** Methodological quality of the included empirical studies (quantitative non-randomized).

reference	Quantitative non-randomized				Total Points	Score
	Low-biased way of recruiting	Measurements appropriate	Consideration of differences between groups	Complete outcome data		
[67]	1	1	1	1	4/4	1.0 (****)
[66]	0	1	1	0	2/4	0.5 (**)
[62]	0	1	1	0	2/4	0.5 (**)
[61]	0	0	1	0	1/4	0.25 (*)
[63]	0	1	1	0	2/4	0.5 (**)
[70]	1	1	1	0	3/4	0.75 (***)
[72]	1	1	1	1	4/4	1.0 (****)

Score (MMAT):

* (25%) = one out of four criteria met;

** (50%) = two out of four criteria met;

*** (75%) = three out of four criteria met;

**** (100%) = all four criteria met

**Table 7:** Methodological quality of the included empirical studies (qualitative).

Reference	Qualitative				Total Points	Score
	Sources of data relevant to objectives	Analysis process relevant to objectives	Consideration of findings relate to context	Consideration of findings relate to researchers’ influence		
[69]	1	1	1	1	4/4	1.0 (****)

Score (MMAT):

**** (100%) = all four criteria met

**Table 8:** Methodological quality of the included empirical studies (mixed methods).

Refe-rence	Quantitative non-randomized				Qualitative				Mixed Methods			Total Points	Score
	Low-biased way of recruiting	Measurements appropriate	Consideration of differences between groups	Complete outcome data	Sources of data relevant to objectives	Analysis process relevant to objectives	Consideration of findings relate to context	Consideration of findings relate to researchers’ influence	Mixed methods research design relevant to objectives	Integration of results relevant to objectives	Consideration of limitations associated with this integration		
[73]	1	0	0	0	1	0	0	0	1	1	1	1/4	0.25 (*)
[64]	1	0	1	0	1	1	1	1	1	1	1	2/4	0.5 (**)

Score (MMAT):

* (25%) = one out of four criteria met;

** (50%) = two out of four criteria met

The recruitment process in four studies [61–63, 66] was potentially affected by selection bias, since the study participants were chosen as a convenience sample. An acceptable response rate was not reached or not clearly identifiable in seven studies [41, 61–64, 66, 73]. Two studies [64, 73] did not precisely explain why they included or excluded eligible participants from the study sample and did not state comprehensibly any reasons for non-participation.

Diggs (2013) applied a mixed-method design in her doctoral thesis with heterogeneous methodological quality scores. The rating of the qualitative part yielded ‘robust’ results, while the quantitative section showed less robust results, since the sample was not representative and the response rate too low. However, the mixed-method approach turned out to be relevant in addressing the research questions and the author identified the limitations that arose from the integration of qualitative and quantitative study findings [64].

### Summary of findings

The study findings were grouped into three themes: 1. acculturation and health, 2. health in the context of discrimination and bullying in the workplace, 3. health in the context of race and ethnic origin.

#### Acculturation and health

Adapting to a foreign country, foreign lifestyles, behaviours and language can lead to stressful experiences among migrant nurses and may affect their health as three studies revealed [61, 66, 73].

Brown and James (2000) described physiological reactions that are due to lifestyle changes. They showed that Filipina nurses with a longer time of residence in their destination country, compared to those with a shorter period, had elevated norepinephrine levels at work and at home as well as a higher diastolic blood pressure during sleep [61]. While a positive correlation between decision latitude and residence time was found among Filipina nurses, neither decision latitude nor psychological demand was significantly associated with blood pressure. Self-reports on how the Filipina nurses’ current lifestyle complied with the Filipino as well as with the American lifestyle, yielded an overall higher mean score for the Filipino lifestyle. These reports were not significantly correlated with blood pressure, any measures of psychological demands, or catecholamine excretion rates [61].

Hener et al. (1997) showed that immigrant nurses and nursing students from the former Soviet Union experienced mild forms of depression during their first months in their host country [66]. After six month they reached normal level. Decreasing levels of depression were explained by growing social support provided by non-family members over time [66]. The lower the students rated the level of perceived social support and satisfaction initially, the higher the level of depression reduction six months later [66].

According to the study of Hayne et al. (2009), Filipino nurses reported feeling distressed due to confusion about their role and a high workload [73]. However, their reported levels of psychological, interpersonal and physical strains were within a normal range. All but one of the Filipino nurses reported doing exercises, having sufficient sleep and typical diets and only two nurses denied receiving excellent social support. Supporting measures within the recruitment and the orientation process helps newly arrived migrant nurses to acculturate in their unknown working and living environment. The authors also showed that migrant nurses who are entitled to practice professional nursing autonomously are satisfied with their job. A positive working environment is enhanced by a positive relationship to working colleagues [73].

#### Health in the context of discrimination and bullying in the workplace

Six studies outlined the relation of discrimination and bullying on the health of migrant nurses [41, 64, 67, 69].

Hogh et al. (2011) showed that immigrant students from Eastern Europe, Iran, Pakistan, Africa or Asia are at significantly higher risk of being bullied in colleges than their native counterparts in Denmark. These non-Western immigrants suffered bullying at their healthcare college and in trainee periods to a much greater extent than natives and twice as often as immigrant students from Iceland, Norway, the 25 EU countries or North America. During their year at college, the risk of being bullied was twice as high for non-Western immigrants compared to native students. While Danish and Western immigrant students did not differ in having been bullied by managers/supervisors or co-workers at follow-up, Western and Non-Western immigrants were significantly more likely to be bullied by clients or residents [67].

Nurses who were recruited by a staffing agency for the US reported significantly lower wages than self-directed foreign nurses and those who received their education in high-income countries [41]. Foreign nurses who reported receiving insufficient orientation and workplace discrimination tended to be unsatisfied with their job. Nurses reported feeling insufficiently orientated concerning their patients’ culture as well the clinical workplace. Foreign nurses who were recruited by a staffing agency experienced far higher levels of perceived discrimination than those recruited directly, whereas different recruitment models did not lead to significant variations in reported job satisfaction among all foreign nurses [41].

A detailed description of experiences of racism, discrimination and bullying can be found in Likupe and Archibong (2013). Black nurses from sub-Saharan Africa working in the United Kingdom report that their experience and knowledge is not taken into account or obviously ignored, not only by White colleagues but also by other overseas nurses, managers, patients and their relatives as well. In the context of discrimination, nurses stated that they were being passed over for promotion and were more likely to be disciplined for mistakes compared to their colleagues. Their requests for days off were often ignored by their supervisors. As a consequence nurses felt considerably distressed, with a possible negative effect on their health as concluded by Likupe and Archibong [69].

Smith Diggs (2013) explained, that male minority nursing students in the USA employ different skills to cope with stress [64]. While the most applied coping skill was ‘planful problem-solving’ (deliberate problem-focused efforts to alter the situation), ‘escape-avoidance’ (wishful thinking and behavioural efforts to escape or avoid the problem) was the coping skill used the least. The minority nursing students reported several physiological, psychological or sociological stressors during their course of studies. The health could be affected by those stressors, as Smith Diggs stated [64].

#### Health in the context of race and ethnic origin

Seven studies examined differences in health-related outcomes among nurses from diverse ethnic backgrounds.

In the study by Brown et al. (1998), Filipino-American nurses reported more frequently negative moods like anxiousness, anger and sadness than Caucasian nurses, who reported more often happiness. At work, both groups of nurses had higher blood pressures than at home. However, Filipino-American nurses’ blood pressure was not elevated while doing household work at home. Caucasian nurses showed a higher increased variance in their diastolic blood pressure while reporting anxiety than did Filipino-American nurses [62]. This ethnic difference did not apply to reports of anger [62]. Compared to Filipino-American nurses, Euro-Americans were significantly more likely to report job strain and higher psychological demands, but showed comparable results for the decision latitude [63]. Nurses who reported having job strain did not show significantly higher values of physiological stress response such as blood pressure or catecholamine excretion rates. Filipino-American and Euro-American nurses also did not differ significantly in their physiological responses to stress in terms of catecholamine excretion, either at home or at work or asleep [63].

Native Yemeni nurses working in public hospitals in Yemen are more frequently affected by low back pain within a year than their migrant colleagues from India. Due to their back pain, they were absent from work for at least one day within one year. Having a higher income, being a non-smoker and having more than an average of five hours of sleep was shown to have a positive but not significant influence on incidence of back pain, while a BMI of more than 25 leads to a higher risk of low back pain within a seven-day time frame [65].

According to Hurtado et al. (2012), Black nurses were at higher risk of reporting job strain than White nurses. Among certified nursing assistants (CNA) the risk for Blacks was three times higher than for Whites. Among higher-skilled nurses, such as Registered or Licensed-Practical nurses (RN/LPN), Black and Whites did not differ from each other in reporting job strain. Black CNAs reported lower control in the context of job strain compared to Whites, while these differences were not detected among the higher-skilled nurses. Black Caribbean nurses reported higher scores than White-Americans, while there was no difference in reporting job strain between African and White-American RNs/LPNs [68].

Simpson and Severson (2000) found that African-American nurses were more likely to report a work-related injury than Whites did. Among the occupations in hospital with frequent risk of injuries, African-American and White nurse’s aides did not differ in having suffered an injury. Among the group of risky occupations, more African-American staff nurses reported an injury compared to Whites. Among the injured nursing staff, there was no difference in the average number of hours worked [70].

Tak et al. (2010) showed that non-Hispanic White nursing assistants (NA) employed in United States nursing homes were more likely to report work-related injuries, while non-Hispanic Black nursing assistants rarely reported back injuries. Non-Hispanic White NAs also more often reported having been bitten by residents. Hispanic NAs had a lower level of education and more non-Hispanic Black NAs reported having a low household income compared to non-Hispanic Whites. Non-Hispanic white NAs were more likely to receive a pay rise than their colleagues from minority racial and ethnic groups [71].

Canadian nurses working in Hawaii experienced higher levels of distress than their colleagues from the Philippines as Beechinor and Fitzpatrick (2008) explained [72]. For Canadian nurses the distress was caused by demands of loss, novelty, and not feeling at home in the receiving country, whereas Philippine nurses reported higher distress due to language accommodation.

Taken together it was shown that the time of residence in the destination country is positively correlated with elevated blood pressure [61]. Findings also revealed that initial depression in the first month after arrival may go into remission over time among migrant nurses [66]. Measures of mental and organizational support turned out to facilitate the process of acculturation for migrant nurses [73]. Migrant nurses, especially those of ethnic and racial minorities, encounter discrimination, racism and bullying at work on a daily basis [67]. This happens in the form of unequal career advancement options, unequal pay, insufficient orientation, overlooking of their skills by colleagues and supervisors [41, 69]. Among migrant nurses of different origins, there are differences in their physiological response to stress [62, 63]. Migrant nurses and native nurses also differ in reporting work-related injuries like back pain [65].

## Discussion

This review provides a comprehensive and rigorous overview of the health of migrant and minority nurses in the international nursing literature to date.

There is a broad range of publications in the context of health of migrant and minority nurses, although only 14 articles focus precisely on the health of those nurses. After getting more familiarized with the body of literature it became apparent that a substantial number of articles do not explicitly discuss the relation between occupational health-related determinants like e.g. job satisfaction, discrimination or social support and health of migrant and minority nurses.

Not only recalling the postulated need for a growing supply of migrant healthcare workers to meet the growing demand for care and treatment—makes it apparent that scholarly interest in this occupational group is becoming established. But also this review supports the trend, that evidence in the context of migrant and minority nurses’ health is emerging. The United States are the dominant employer of migrant and minority nurses and a majority of the research has been conducted there. New Zealand, Switzerland, Australia and Luxembourg also constitute a major market for migrant nurses within the OECD, but studies that have been conducted in these countries are scarce.

Most of the migrant nurses within the OECD originate from the Philippines and India. Besides these nurses, this review also identified studies that observed nurses from various other countries around the world like South America, the former Soviet Union, Yemen, and Israel.

In the context of acculturation and health it was discussed whether longer periods of residence in the host country lead to either an attenuated stress response or to increased levels in terms of elevated blood pressure or higher levels of depression [61, 66]. Lee et al. (2016) observed that a lower ten-year cardiovascular disease risk in Korean-Chinese migrant female workers compared to native Koreans was due to the healthy migrant effect. Korean-Chinese immigrant workers’ health decreases to the same level as that of natives, due to being exposed to unhealthy lifestyles and working conditions [74]. Delavari et al. (2013) stated that the health migrant effect may diminish with increasing acculturation in high-income countries due to adapting to unhealthy eating habits of the host culture. [75]. Having said that, ongoing acculturation to the host culture can lead to health-related problems and thus, acculturation programs should also emphasise on retaining the positive parts of the traditional orientation and combine them with the positive aspects of the host culture [75].

Lee et al. (2016) endorsed the need for implementing health information and behaviours, particularly in the early migration phase to raise migrant workers’ awareness and knowledge of cardiovascular disease prevention [74]. The positive effect of supporting measures in the early phase of migration on the well-being of migrant nurses was indicated by Hayne et al. (2009). They explained that supporting measures within the recruitment and orientation process helps newly arrived migrant nurses to acculturate in their unknown working and living environment [73].

The health of migrant nurses, especially those of ethnic and racial minorities, is also affected by experiences of discrimination and bullying at work, as this review identified. Paradies et al. (2015) proved that racism is significantly related to poorer health, and that this correlation is stronger in relation to poor mental health and weaker in relation to poor physical health [76]. Pung and Goh (2016) confirmed the patterns of discrimination towards migrant nurses and explained that discrimination can lead to impaired job performance and increased stress levels. Discrimination against migrant nurses affects staff morale and results in high turnover rates. Migrant nurses who experience discrimination and racism lack the confidence to report these incidences since they are frightened of isolation and retaliation [77]. Garner et al. (2015) explained that if the migrant nurses’ residency status depends on their employment status, they may be less likely to report discrimination or criticize colleagues [78].

The need for transition programmes that include cultural training for migrant nurses, as well as for their host colleagues, has been confirmed in several studies. These programmes should promote cultural competency and thus prevent perceptions and experiences of discrimination and unfair practices [78–80]. However, the awareness for the need of cultural competent transition programmes still stands in the need of development [26, 81]. An effective cultural competence model should be based on four central components: professional level, individual level, systematic and organisational level [26]. Whether those programmes effectively allow migrant nurses and their host colleagues an adequate interacting at the workplace still remains unclear.

Nurses from poorer countries with lower compensation rates are encouraged to leave their home country to receive more attractive career opportunities and higher compensation [6, 17]. There is evidence that migrant nurses receive higher compensation in their host country [82], while this review revealed that migrant nurses face discrimination in terms of having not received a pay rise, even if they were entitled to it [71]. Being passed over for promotion or professional training [40] represents a further kind of reported discrimination.

Migrants and non-native ethnic groups experience poorer health outcomes like accidents or disabilities than natives [19]. Several studies show higher prevalence rates for psychiatric disorders among migrants compared to the majority population [25, 83, 84]. These findings are also present in this review. However, reported negative moods, work-related injuries or job strain differs across ethnicity: even though nurses have the same occupational background, share the same working conditions and do the same tasks, their reporting behaviour differs across ethnic and racial groups [62, 65, 70, 71]. Self-reporting bias can affect reported outcomes as Akresh (2008) and Stevens et al. (2003) [85, 86] discussed. They stated that people vary strongly in reporting moods or incidents. Social desirability as well as cultural influences can lead to the tendency to underreport incidents. This could explain the divergent reporting behaviour among migrant and native nurses in the context of occupational injuries and impairments. Differences in ‘ethnocultural standards’ [86] can also cause differences in self-reporting and thus explain why nurses from different countries of origin and with different racial and ethnic backgrounds experience their working conditions differently.

In terms of methodological quality of the studies, five studies met all four criteria and 3 studies passed 3 criteria according to MMAT. Six studies showed weaker values as four just met two criteria and the remaining two reached one criteria (Tables 5 to 8). Convenience sampling and a low response rate turned out to be the major challenges. The applied study instruments were mainly self-developed and not validated. Many psychological instruments were developed and standardized based on a primarily Caucasian population and thus they were inappropriate to be applied to minority groups [87, 88]. Six of the studies [61, 63, 64, 66, 68, 72, 73] that were included in the final summary employed four validated instruments in total (Tables 1 to 4). Three instruments were successfully tested for cross-cultural validity, whereas the Beck Depression Inventory was tested among African-American and Caucasian students [88], the Job Content Questionnaire among Malay nurses [89], Iranian healthcare workers [90, 91] and hospital workers in Taiwan [92]. The same applies to the Nurses Work Index, which was validated for hospital nurses in South Korea [93] and Asian nurses working in the US [94]. However, it was unclear whether a culturally appropriate version of these three questionnaires was applied within the five studies in the final summary.

### Strengths and limitations

Articles in languages other than English and German have not been obtained for abstract screening. Therefore some articles of interest published in other languages could be missing in this review. The abstract screening was performed by two persons, but one person screened three quarters of the titles. The data extraction was performed by the main author, but cross-checked by a further member of the research group.

This systematic review also has its distinct strengths, since a comprehensive literature search including recent and unpublished articles or theses has been performed. A date restriction for the database searches was not set. As a consequence, a broad extent of literature provided the basis for this review. The search terms covered a very broad topical range in the context of health, nurses and migration. Finally, a methodological quality assessment has been applied to support the findings of the study.

## Conclusion

There are just 14 articles that explicitly focus on migrant and minority nurses’ health in the international research literature. These studies agree that migrant and minority nurses are at a higher risk of work-related injuries and discrimination than native or majority ethnicity nurses. However, mixed results were obtained. The reported health of migrant nurses can improve over time or decrease. Different patterns of behaviour are possibly due to different ‘ethnocultural standards’. The study results rather indicate that migrant and minority nurses and native nurses do not differ in reported health outcomes while sharing similar working conditions, except for experiences of discrimination or bullying. It is evident that racism is likely to be a health risk for the victim.

Further comparative studies with strong methodological quality and standardized outcome measures are needed to support and further establish the evidence found in existing studies. Longitudinal studies are required to determine the success of acculturation processes for newly arrived migrant nurses. Health-related self-reports of subjects such as migrant and minority nurses may lead to over- or underreporting of health outcomes. Even though cross-cultural versions of standard measurements are available, there is a further need to design more. Direct measures like medical screenings should also be employed more frequently to assess the health of migrant and minority nurses, since direct measures are less affected by reporting bias than self-reports on health. More research on migrant and minority nurses’ health should also be conducted in European countries. There is also a need for further insight into the wellbeing of migrant nurses’ originating from countries other than the Philippines.

Recruiting migrant healthcare workers is a key topic on the international political agenda [95, 96]. In order to successfully recruit and retain migrant nurses policymakers, organizations and those responsible at meso- and micro levels are requested to provide them a healthy workplace. This systematic review also revealed that discrimination is a major cause of impaired health for minority nurses. Thus, awareness of this danger should be raised.

## Supporting information

S1 Appendix Entire search strings for databases (Medline, PsycINFO, CINAHL and Web of Science). (DOCX)

S2 Appendix List of excluded full texts. (XLSX)

S3 Appendix Detailed study characteristics of included studies. (XLSX)

S4 Appendix PRISMA-Checklist. (DOCX)
