# Supplementary material for: Health situation of migrant and minority nurses: A systematic review
Source: PLoS One. 2017 Jun 26;12(6):e0179183. doi: 10.1371/journal.pone.0179183 (PMC5484487; doi:10.1371/journal.pone.0179183)
Supplement: S1 Appendix — (DOCX) [file pone.0179183.s001.docx]

**Search strategy in Medline via Pubmed**

1. Health OR Determinants of Health
2. Nurse AND Migration AND 1
3. Migrant Nurse AND 1
4. 2 NOT 3
5. 3 NOT 2
6. Limit 4 to Abstracts available/ Human/ English/ German
7. Limit 5 to Abstracts available/ Human/ English/ German

**Health**

"health"[Mesh] OR "health"[ALL] OR "Health Status"[Mesh] OR ("health"[ALL] AND "status"[All]) OR "health status"[ALL] OR "mental health"[Mesh] OR "mental health"[ALL] OR ("mental"[ALL] AND "health"[ALL]) OR "Occupational Health"[Mesh] OR "Occupational Health"[ALL] OR ("Occupational"[ALL] AND "Health"[ALL]) OR "Health Behavior"[Mesh] OR "Health Behavior"[ALL] OR ("Health"[ALL] AND "Behavior"[ALL]) R "Health Behaviour"[ALL] OR ("Health"[ALL] AND "Behaviour"[ALL]) OR "ill health"[ALL] OR ("ill"[ALL] AND "health"[ALL]) OR "Disease"[Mesh] OR "Disease"[ALL] OR "Diseases"[ALL])

**Determinants of Health**"professional satisfaction"[ALL] OR ("professional"[ALL] AND "satisfaction"[ALL]) OR "well- being"[ALL] OR ("well"[ALL] AND "being"[ALL]) OR "Job Satisfaction"[Mesh] OR "Job Satisfaction"[ALL] OR ("Job"[ALL] AND "Satisfaction"[ALL]) OR "Personal Satisfaction"[Mesh] OR "Personal Satisfaction"[ALL] OR ("Personal"[ALL] AND "Satisfaction"[ALL]) OR "Quality of Life"[Mesh] OR "Quality of Life"[ALL] OR ("Quality"[ALL] AND "of"[ALL] AND "Life"[ALL]) OR "job control"[ALL] OR ("job"[ALL] AND "control"[ALL]) OR "responsibility"[ALL] OR "autonomy"[ALL] OR "effort"[ALL] OR "reward"[Mesh] OR "reward"[ALL] OR "team work"[ALL] OR ("team"[ALL] AND "work"[ALL]) OR "Acculturation"[Mesh] OR "Acculturation"[ALL] OR "mortality"[Mesh] OR "mortality"[ALL] OR "morbidity"[Mesh] OR "morbidity"[ALL] OR "Occupational Diseases"[Mesh] OR "Occupational Diseases"[ALL] OR "Occupational Disease"[ALL] OR ("Occupational"[ALL] AND "Diseases"[ALL]) OR ("Occupational"[ALL] AND "Disease"[ALL]) OR "Occupational Exposure"[Mesh] OR "Occupational Exposure"[ALL] OR "Occupational Exposures"[ALL] OR ("Occupational"[ALL] AND "Exposure"[ALL]) OR ("Occupational"[ALL] AND "Exposures"[ALL]) OR "Mental Disorders"[Mesh] OR "Mental Disorders"[ALL] OR "Mental Disorder"[ALL] OR ("Mental"[ALL] AND "Disorders"[ALL]) OR ("Mental"[ALL] AND "Disorder"[ALL]) OR "mental illness"[ALL] OR "mental illnesses"[ALL] OR ("mental"[ALL] AND "illness"[ALL]) OR ("mental"[ALL] AND "illnesses"[ALL]) OR "Stress, Psychological"[Mesh] OR "Stress, Psychological"[ALL] OR ("Stress"[ALL] AND "Psychological"[ALL]) OR "Stress, Physiological"[Mesh] OR "Stress, Physiological"[ALL] OR ("Stress"[ALL] AND "Physiological"[ALL]) OR "Stress Disorders, Traumatic"[Mesh] OR "Stress Disorders, Traumatic"[ALL] OR "Stress Disorder, Traumatic"[ALL] OR ("Stress"[ALL] AND "Disorders"[ALL] AND "Traumatic"[ALL]) OR ("Stress"[ALL] AND "Disorder"[ALL] AND "Traumatic"[ALL]) OR "Anxiety"[Mesh] OR "Anxiety"[ALL] OR "Depression"[Mesh] OR "Depression"[ALL] OR "Burnout, Professional"[Mesh] OR "Burnout, Professional"[ALL] OR ("Burnout"[ALL] AND "Professional"[ALL]) OR

"Fatigue"[Mesh] OR "Fatigue"[ALL] OR "Resilience, Psychological"[Mesh] OR "Resilience, Psychological"[ALL] OR ("Resilience"[ALL] AND "Professional"[ALL]) OR "Absenteeism"[Mesh] OR "Absenteeism"[ALL] OR "Work Schedule Tolerance"[Mesh] OR "Work Schedule Tolerance"[ALL] OR ("Work"[ALL] AND "Schedule"[ALL] AND "Tolerance"[ALL]) OR "Personnel Turnover"[Mesh] OR "Personnel Turnover"[ALL] OR ("Personnel"[ALL] AND "Turnover"[ALL]) OR "double employment"[ALL] OR ("double"[ALL] AND "employment"[ALL]) OR "dual practice"[ALL] OR ("dual"[ALL] AND "practice"[ALL]) OR "Low Back Pain"[Mesh] OR "Low Back Pain"[ALL] OR ("Low"[ALL] AND "Back"[ALL] AND "Pain"[ALL]) OR "Musculoskeletal Diseases"[Mesh] OR "Musculoskeletal Diseases"[ALL] OR "Musculoskeletal Disease"[ALL] OR ("Musculoskeletal"[ALL] AND "Diseases"[ALL]) OR ("Musculoskeletal"[ALL] AND "Disease"[ALL]) OR "Musculoskeletal Pain"[Mesh] OR "Musculoskeletal Pain"[ALL] OR "Musculoskeletal Pains"[ALL] OR ("Musculoskeletal"[ALL] AND "Pains"[ALL]) OR ("Musculoskeletal"[ALL] AND "Pain"[ALL]) OR "Needlestick Injuries"[Mesh] OR "Needlestick Injuries"[ALL] OR "Needlestick Injury"[ALL] OR ("Needlestick"[ALL] AND "Injuries"[ALL]) OR ("Needlestick"[ALL] AND "Injury"[ALL]) OR "Sleep"[Mesh] OR "Sleep"[ALL] OR "Sleep Arousal Disorders"[Mesh] OR "Sleep Arousal Disorders"[ALL] OR "Sleep Arousal Disorder"[ALL] OR ("Sleep"[ALL] AND "Arousal"[ALL] AND "Disorders"[ALL]) OR ("Sleep"[ALL] AND "Arousal"[ALL] AND "Disorder"[ALL]) OR "Sleep Disorders, Intrinsic"[Mesh] OR "Sleep Disorders Intrinsic"[ALL] OR ("Sleep"[ALL] AND "Disorders"[ALL] AND "Intrinsic"[ALL]) OR ("Sleep"[ALL] AND "Disorder"[ALL] AND "Intrinsic"[ALL]) OR "Neoplasms"[Mesh] OR "Neoplasms"[ALL] OR "Neoplasm"[ALL] OR "Tuberculosis"[Mesh] OR "Tuberculosis"[ALL] OR "HIV Infections"[Mesh] OR "HIV Infections"[ALL] OR "HIV Infection"[ALL] OR ("HIV"[ALL] AND "Infections"[ALL]) OR ("HIV"[ALL] AND "Infection"[ALL]) OR "Cytomegalovirus"[Mesh] OR "Cytomegalovirus"[ALL] OR "CMV"[ALL] OR "Hepatitis"[Mesh] OR "Hepatitis"[ALL] OR "Hypersensitivity"[Mesh] OR "Hypersensitivity"[ALL] OR "Allergy and Immunology"[Mesh] OR "Allergy and Immunology"[ALL] OR ("Allergy"[ALL] AND "Immunology"[ALL]) OR "Diabetes Mellitus"[Mesh] OR "Diabetes Mellitus"[ALL] OR ("Diabetes"[ALL] AND "Mellitus"[ALL]) OR "Hypertension"[Mesh] OR "Hypertension"[ALL] OR "Smoking"[Mesh] OR "Smoking"[ALL] OR "Alcohol Drinking"[Mesh] OR "Alcohol Drinking"[ALL] OR ("Alcohol"[ALL] AND "Drinking"[ALL]) OR "Substance-Related Disorders"[Mesh] OR "Substance-Related Disorders"[ALL] OR "Substance-Related Disorder"[ALL] OR ("Substance"[ALL] AND "Related"[ALL] AND "Disorders"[ALL]) OR ("Substance"[ALL] AND "Related"[ALL] AND "Disorder"[ALL])

**Nurse**

"Nursing Staff"[Mesh] OR "Nursing Staff"[ALL] OR ("Nursing"[ALL] AND "Staff"[ALL]) OR "Nurses"[Mesh] OR "Nurses"[ALL] OR "Nurse"[ALL] OR "Nurses' Aides"[Mesh] OR "Nurses' Aides"[ALL] OR "Nurses' Aide"[ALL] OR ("Nurses"[ALL] AND "Aides"[ALL]) OR ("Nurses"[ALL] AND "Aide"[ALL]) OR "nurse aide"[ALL] OR "nurse aides"[ALL] OR ("nurse"[ALL] AND "aide"[ALL]) OR ("nurse"[ALL] AND "aides"[ALL]) OR "Students, Nursing"[Mesh] OR "Student, Nursing"[ALL] OR "Students, Nursing"[ALL] OR ("Nursing"[ALL] AND "Student"[ALL]) OR ("Nursing"[ALL] AND "Students"[ALL]) OR "nursing assistant"[ALL] OR "nursing assistants"[ALL] OR ("nursing"[ALL] AND "assistant"[ALL]) OR ("nursing"[ALL] AND "assistants"[ALL]) OR "auxiliary nurse"[ALL] OR "auxiliary nurses"[ALL] OR ("auxiliary"[ALL] AND "nurse"[ALL]) OR ("auxiliary"[ALL] AND "nurses"[ALL]) OR "homecare workers"[ALL] OR "homecare worker"[ALL] OR ("homecare"[ALL] AND "workers"[ALL]) OR ("homecare"[ALL] AND "worker"[ALL]) OR "live-in caregivers"[ALL] OR "live-in caregiver"[ALL] OR ("live-in"[ALL] AND "caregivers"[ALL]) OR ("live-in"[ALL] AND "caregiver"[ALL]) OR "care worker"[ALL] OR "care workers"[ALL] OR ("care"[ALL] AND "worker"[ALL]) OR ("care"[ALL] AND "workers"[ALL]) OR "home attendants"[ALL] OR "home attendant"[ALL] OR ("home"[ALL] AND "attendants"[ALL]) OR ("home"[ALL] AND "attendant"[ALL] OR "healthcare aide"[ALL] OR "healthcare aides"[ALL] OR ("healthcare"[ALL] AND "aide"[ALL]) OR ("healthcare"[ALL] AND "aides"[ALL]) OR "Home Health Aides"[Mesh] OR "Home Health Aides"[ALL] OR "Home Health Aide"[ALL] OR ("Home"[ALL] AND "Health"[ALL] AND "Aides"[ALL]) OR ("Home"[ALL] AND "Health"[ALL] AND "Aide"[ALL]) OR "Home Nurses"[ALL] OR "Home Nurse"[ALL] OR ("Home"[ALL] AND "Nurses"[ALL]) OR ("Home"[ALL] AND "Nurse"[ALL]) OR "Homecare worker"[ALL] OR "Homecare workers"[ALL] OR ("Homecare"[ALL] AND "worker"[ALL]) OR ("Homecare"[ALL] AND "workers" [ALL]) OR "home health workers"[ALL] OR "home health worker"[ALL] OR ("home"[ALL] AND "health"[ALL] AND "workers"[ALL]) OR ("home"[ALL] AND "health"[ALL] AND "worker"[ALL]) OR "Caregivers"[Mesh] OR "Caregivers"[ALL] OR "Caregiver"[ALL] NOT "parents"[ALL] NOT ("spouse"[ALL] OR "spouses"[ALL]) NOT "family members"[ALL]

**Migration and Minority**

"Human Migration"[Mesh] OR "Human Migration"[ALL] OR ("Human"[ALL] AND "Migration"[ALL]) OR "Transients and Migrants"[Mesh] OR "Transients and Migrants"[ALL] OR "Transient and Migrant"[ALL] OR ("Transients"[ALL] AND "Migrants"[ALL]) OR ("Transient"[ALL] AND "Migrant"[ALL]) OR "Emigration and Immigration"[Mesh] OR "Emigration and Immigration"[ALL] OR ("Emigration"[ALL] AND "Immigration"[ALL]) OR "ethnicity"[ALL] OR "Ethnic Groups"[Mesh] OR "Ethnic Groups"[ALL] OR ("Ethnic" [ALL] AND "Groups"[ALL]) OR "Ethnic Group"[ALL] OR ("Ethnic" [ALL] AND "Group"[ALL]) OR
"Ethnology"[Mesh] OR "Ethnology"[ALL] OR "workforce migration"[ALL] OR ("workforce"[ALL] AND "migration"[ALL]) OR "nurse migration"[ALL] OR ("nurse"[ALL] AND "migration"[ALL])

**Migrant and Minority Nurse**"foreign-born nurses"[ALL] OR ("foreign-born"[ALL] AND "nurses"[ALL]) OR ("foreign-born"[ALL] AND "nurse"[ALL]) OR "foreign domestic workers"[ALL] OR ("foreign"[ALL] AND "domestic"[ALL] AND "worker"[ALL]) OR ("foreign"[ALL] AND "domestic"[ALL] AND "workers"[ALL]) OR ("migrant"[ALL] AND "live-in"[ALL] AND "caregivers"[ALL]) OR ("migrant"[ALL] AND "live-in"[ALL] AND "caregiver"[ALL]) OR "migrant domestic workers"[ALL] OR ("migrant"[ALL] AND "domestic"[ALL] AND "worker"[ALL]) OR ("migrant"[ALL] AND "domestic"[ALL] AND "workers"[ALL]) OR "migrant care worker"[ALL] OR "migrant care workers"[ALL] OR ("migrant"[ALL] AND "care"[ALL] AND "worker"[ALL]) OR ("migrant"[ALL] AND "care"[ALL] AND "workers"[ALL]) OR migra* nurse[ALL] OR migra* nurses[all] OR (migra* AND nurse[ALL]) OR (migra* AND nurses[ALL]) OR immigra* nurse[ALL] OR immigra* nurses[ALL] OR (immigra* AND nurse[ALL]) OR (immigra* AND nurses[ALL]) OR "Philippine Nurses"[ALL] OR "Philippine Nurses"[ALL] OR ("Philippine"[ALL] AND "Nurses"[ALL]) OR ("Philippine"[ALL] AND "Nurse"[ALL]) OR "Filipino Nurses"[ALL] OR "Filipino Nurse"[ALL] OR ("Filipino"[ALL] AND "Nurses"[ALL]) OR ("Filipino"[ALL] AND "Nurse"[ALL]) OR "Indian Nurses"[ALL] OR "Indian Nurse"[ALL] OR ("Indian"[ALL] AND "Nurses"[ALL]) OR ("Indian"[ALL] AND "Nurse"[ALL]) OR "African Nurses"[ALL] OR "African Nurse"[ALL] OR ("African"[ALL] AND "Nurses"[ALL]) OR ("African"[ALL] AND "Nurse"[ALL]) OR "ethnic nurses"[ALL] OR ("ethnic"[ALL] AND "nurse"[ALL]) OR ("ethnic"[ALL] AND "nurses"[ALL]) OR

"Overseas nurse"[ALL] OR "Overseas nurses"[ALL] OR ("Overseas"[ALL] AND "nurse"[ALL]) OR ("Overseas"[ALL] AND "nurses"[ALL]) OR "foreign nurse"[ALL] OR "foreign nurses"[ALL] OR ("foreign"[ALL] AND "nurse"[ALL]) OR ("foreign"[ALL] AND "nurses"[ALL])

**Search strategy in CINAHL**

1. Nurse AND Migration AND Health
2. Migrant Nurse AND Health
3. 1 NOT 2
4. 2 NOT 1
5. Limit 3 to Abstracts available/ English
6. Limit 4 to Abstracts available/English

**Nurse**

S1:(MH "Nursing Staff, Hospital") OR (MH "Nursing Home Personnel") OR (MH "Staff Nurses")

S2:(MH "Nurses+") OR (MH "Practical Nurses")

S3:(MH "Nursing Assistants")

S4:(MH "Students, Nursing, Male") OR (MH "Students, Nursing, Graduate") OR (MH "Students, Nursing, Associate") OR (MH "Students, Post-RN") OR (MH "Students, Nursing, Practical") OR (MH "Students, Pre-Nursing") OR (MH "Students, Nursing") OR (MH "Students, Nursing, Baccalaureate") OR (MH "Students, Nursing, Diploma Programs") OR (MH "Students, Nursing, Masters") OR (MH "Students, Nursing, Doctoral")

S5:(MH "Home Health Aides")

S6:(MH "Shift Workers") OR (MH "Caregivers") OR (MH "Workforce")

S7:TX auxiliary nurse*

S8:TX home care worker*

S9:TX live-in caregiver*

S10:TX care worker*

S111:home attendant

S12:TX health care aide*

S13:TX home nurse*

S14: S1 OR S2 OR S3 OR S4 OR S5 OR S6 OR S7 OR S8 OR S9 OR S10 OR S11 OR S12 OR S13

**Migration and Minority**

S1:(MH "Transients and Migrants")

S2:(MH "Emigration and Immigration")

S3:(MH "Ethnic Groups+")

S4:TX Human Migration

S5: S1 OR S2 OR S3 OR S4

**Health**

S1:(MH "Health+")

S2:(MH "Health Status+")

S3:(MH "Mental Health")

S4:(MH "Occupational Health+")

S5:(MH "Health Behavior+")

S6:(MH "Disease+")

S7:TX ill health

S8:(MH "Job Satisfaction+")

S9:(MH "Personal Satisfaction+")

S10:(MH "Quality of Life+") OR (MH "Quality of Working Life")

S11:(MH "Reward")

S12:(MH "Acculturation")

S13:(MH "Mortality+")

S14:(MH "Morbidity+")

S15:(MH "Occupational Diseases+")

S16:(MH "Occupational Exposure")

S17:(MH "Mental Disorders+")

S18:(MH "Stress, Psychological+")

S19:(MH "Stress, Physiological")

S20:(MH "Stress Disorders, Post-Traumatic+")

S21:(MH "Anxiety+")

S22:(MH "Depression+")

S23:(MH "Burnout, Professional")

S24:(MH "Fatigue+") OR (MH "Mental Fatigue")

S25:(MH "Hardiness")

S26:(MH "Personnel Staffing and Scheduling+")

S27:(MH "Back Pain+") OR (MH "Low Back Pain")

S28:(MH "Musculoskeletal Diseases+")

S29:(MH "Needlestick Injuries") OR (MH "Occupational-Related Injuries")

S30:(MH "Sleep+") OR (MH "Sleep Disorders, Circadian Rhythm+") OR (MH "Sleep Disorders+") OR (MH "Sleep Arousal Disorders+")

S31:(MH "Neoplasms+")

S32:(MH "Tuberculosis+")

S33:(MH "Human Immunodeficiency Virus+")

S34:(MH "Cytomegalovirus Infections+") OR (MH "Cytomegaloviruses")

S35:(MH "Cytomegalovirus Infections+") OR (MH "Cytomegaloviruses")

S36:(MH "Hypersensitivity+")

S37:(MH "Allergy and Immunology")

S38:(MH "Diabetes Mellitus+")

S39:(MH "Hypertension+")

S40:(MH "Smoking+")

S41:(MH "Alcohol Drinking+")

S42:(MH "Substance Use Disorders+")

S43:TX well-being

S44:TX job control

S45:TX responsibility

S46:TX autonomy

S47:TX effort

S48:TX team work*

S49:TX absenteeism

S50:TX dual practice

S51: S1 OR S2 OR S3 OR S4 OR S5 OR S6 OR S7 OR S8 OR S9 OR S10 OR S11 OR S12 OR S13 OR S14 OR S15 OR S16 OR S17 OR S18 OR S19 OR S20 OR S21 OR S22 OR S23 OR S24 OR S25 OR S26 OR S27 OR S28 OR S29 OR S30 OR S31 OR S32 OR S33 OR S34 OR S35 OR S36 OR S37 OR S38 OR S39 OR S40 OR S41 OR S42 OR S43 OR S44 OR S45 OR S46 OR S47 OR S48 OR S49 OR S50

**Migrant and Minority Nurse**

S1:(MH "Foreign Nurses")

S2:TX foreign domestic workers

S3:TX migrant nurs*

S4:TX foreign domestic worker

S5:TX migrant caregivers

S6:TX migrant care worker

S7:TX immigrant nurs*

S8:TX immigrant caregiver

S9:TX Philippine nurs*

S10:TX Filipino nurs*

S111:TX indian nurs*

S12:TX african nurs*

S13:TX ethnic nurs*

S14:TX overseas nurs*

S15:(MH "Health Personnel, Minority+")

S16:(MH "Health Personnel, Minority+") OR (MH "Nurses, Minority")

S17: S1 OR S2 OR S3 OR S4 OR S5 OR S6 OR S7 OR S8 OR S9 OR S10 OR S11 OR S12 OR S13 OR S14 OR S15 OR S16

**Search strategy in PsychInfo**

1. Nurse AND Migration AND Health
2. Migrant Nurse AND Health
3. 1 NOT 2
4. 2 NOT 1
5. Limit 3 to Abstracts available/ Human/ English/ German
6. Limit 4 to Abstracts available/ Human/ English/ German

**Nurse**

1. exp Psychiatric Nurses/ or exp Nurses/ or nursing staff.mp.

2. exp Nursing Students/ or nursing student*.mp.

3. exp Home Care Personnel/

4. homecare worker.mp.

5. health?care aid*.mp.

6. home health worker*.mp.

7. nursing aide*.mp.

8. nursing assistant*.mp.

9. care?worker*.mp.

10. home attendant*.mp.

11. Home health aide*.mp.

12. exp Family Members/

13. exp Caregivers/

14. 13 not 12

15. 1 or 2 or 3 or 4 or 5 or 6 or 7 or 8 or 9 or 10 or 11 or 14

**Migration and Minority**

1. exp Human Migration/

2. exp Ethnic Identity/ or ethnic.mp.

3. exp Ethnic Values/

4. exp Ethnology/

5. nurse migration.mp.

6. workforce migration.mp.

7. exp Immigration/

8. 1 or 2 or 3 or 4 or 5 or 6 or 7

**Health**

1. exp Health/ or health.mp.
2. Health Status.mp.

3. mental health.mp. or exp Mental Health/

4. occupational health.mp. or exp Occupational Health/

5. health behavior.mp. or exp Health Behavior/

6. ill health.mp.

7. disease.mp.

8. exp Satisfaction/ or exp Job Satisfaction/ or professional satisfaction.mp.

9. personal satisfaction.mp.

10. well being.mp. or exp Well Being/

11. quality of life.mp. or exp "Quality of Life"/
12. job control.mp.
13. responsibility.mp. or exp Responsibility/
14. exp Self Determination/ or autonomy.mp.
15. effort.mp.
16. exp Rewards/ or reward.mp.
17. exp Group Dynamics/ or exp Group Performance/ or team work.mp.
18. leadership.mp. or exp Leadership/ or exp Leadership Style/
19. acculturation.mp. or exp Acculturation/
20. exp Cross Cultural Differences/
21. mortality.mp. or exp "Death and Dying"/
22. morbidity.mp. or exp Morbidity/
23. exp Disorders/ or exp Work Related Illnesses/ or exp Occupational Stress/ or exp Occupational Exposure/ or occupational disease.mp. or exp Disabilities/
24. mental disorder.mp. or exp Mental Disorders/
25. mental illness.mp.
26. exp Physiological Stress/ or exp Psychological Stress/ or exp Chronic Stress/ or exp Occupational Stress/ or exp Posttraumatic Stress Disorder/ or STress.mp. or exp Stress/
27. exp Distress/ or distress.mp.
28. exp Anxiety/ or anxiety.mp.
29. depression.mp. or exp Recurrent Depression/ or exp Major Depression/ or exp Treatment Resistant Depression/ or exp "Depression (Emotion)"/
30. burnout.mp.
31. exp Fatigue/ or fatigue.mp.
32. exp Coping Behavior/ or exp "Resilience (Psychological)"/ or exp Psychological Endurance/ or resilience.mp.
33. exp Employee Turnover/ or exp Employee Absenteeism/ or exp Job Performance/ or absenteeism.mp. or exp Motivation/
34. exp Family Work Relationship/ or work schedule.mp.
35. double employment.mp.
36. dual practice.mp.
37. exp Back Pain/ or low* back pain.mp.
38. exp Musculoskeletal Disorders/ or musculoskeletal disease.mp.
39. musculoskeletal pain.mp.
40. exp Injuries/
41. exp Workers' Compensation Insurance/
42. exp Sleep Deprivation/ or exp Sleep/ or exp Sleep Disorders/
43. neoplasm.mp. or exp Breast Neoplasms/ or exp Benign Neoplasms/ or exp Brain Neoplasms/ or exp Nervous System Neoplasms/ or exp Neoplasms/ or exp Endocrine Neoplasms/
44. exp Tuberculosis/ or tuberculosis.mp.
45. exp HIV/
46. exp Infectious Disorders/ or exp Health Impairments/ or exp Viral Disorders/ or exp Congenital Disorders/
47. exp Hepatitis/
48. exp Pain/ or exp Immunologic Disorders/ or hypersensitivity.mp.
49. exp Allergic Disorders/ or allergy.mp.
50. allergies.mp.
51. Diabetes.mp. or exp Diabetes/ or exp Diabetes Mellitus/

52. exp Hypertension/ or exp Essential Hypertension/ or hypertension.mp.
53. smoking.mp. or exp Passive Smoking/ or exp Tobacco Smoking/
54. exp Alcohol Intoxication/ or exp Alcohol Drinking Patterns/ or exp Driving Behavior/
55. exp Drug Abuse/ or exp Anxiety/ or exp Alcohol Abuse/
56. 1 or 2 or 3 or 4 or 5 or 6 or 7 or 8 or 9 or 10 or 11 or 12 or 13 or 14 or 15 or 16 or 17 or 18 or 19 or 20 or 21 or 22 or 23 or 24 or 25 or 26 or 27 or 28 or 29 or 30 or 31 or 32 or 33 or 34 or 35 or 36 or 37 or 38 or 39 or 40 or 41 or 42 or 43 or 44 or 45 or 46 or 47 or 48 or 49 or 50 or 51 or 52 or 53 or 54 or 55

**Migrant and Minority Nurses**

1. foreign nurse*.mp.

2. foreign domestic worker*.mp.

3. migrant care worker*.mp.

4. migrant caregiver*.mp.

5. migra$ nurse*.mp.

6. immigra$ nurse*.mp.

7. philippine nurse*.mp.

8. filipin* nurse*.mp.

9. indian nurse*.mp.

10. african nurse*.mp.

11. ethnic nurse*.mp.

12. oversea* nurse*.mp.

13. 1 or 2 or 3 or 4 or 5 or 6 or 7 or 8 or 9 or 10 or 11 or 12

**Search strategy in Web of Science**

1. Health OR Determinants of Health
2. Nurse AND Migration AND 1
3. Migrant Nurse AND 1
4. 2 NOT 3
5. 3 NOT 2

**Health**

TS=health OR TS="health status" OR TS="mental health" OR TS="Occupational Health" OR TS="Health Behavior" OR TS="ill health" OR TS=Disease

**Determinants of Health**

TS="professional satisfaction" OR TS="well being" OR TS="Job Satisfaction" OR TS="Personal Satisfaction" OR TS="Quality of Life" OR TS="job control" OR TS=responsibility OR TS=autonomy OR TS=effort OR TS=reward OR TS="team work" OR TS="Acculturation" OR TS=mortality OR TS=morbidity OR TS="Occupational Disease" OR TS="Occupational Exposure" OR TS="Mental Disorders" OR TS="mental illness" OR TS="Psychological Stress" OR TS="Physiological Stress" OR TS="Stress Disorders" OR TS="Stress Disorder" OR TS=Anxiety OR TS=Depression OR TS=Burnout OR TS=Fatigue OR TS=Resilience OR TS=Absenteeism OR TS="double employment" OR TS="dual practice" OR TS="Back Pain" OR TS="Musculoskeletal Diseases" OR TS="Musculoskeletal Pain" OR TS="Needlestick Injuries" OR TS="Needlestick Injury" OR TS=Sleep OR TS=Neoplasm* OR TS=cancer OR TS=Tuberculosis OR TS=HIV OR TS=Cytomegalovirus OR TS=Hepatitis OR TS=Hypersensitivity OR TS=Allergy OR TS=Allergies OR TS="Diabetes Mellitus" OR TS=Diabetes OR TS=Hypertension OR TS="blood pressure" OR TS=Smoking OR TS="Alcohol Drinking" OR TS="Substance Abuse"

**Nurses**

TS="Nursing Staff" OR TS=Nurse* OR TS="Nurses' Aides" OR TS="Nurses' Aide" OR TS="Nursing Student" OR TS="Nursing Students" OR TS="nursing assistant" OR TS="nursing assistants" OR TS="auxiliary nurse" OR TS="auxiliary nurses" OR TS="homecare workers" OR TS="homecare worker" OR TS="live-in caregivers" OR TS="live-in caregiver" OR TS="care worker" OR TS="care workers" OR TS="home attendants" OR TS="home attendant" OR TS="healthcare aide" OR TS="healthcare aides" OR TS="Home Health Aide" OR TS="Home Health Aides" OR TS="Home Nurses" OR TS="Home Nurse" OR TS="Homecare worker" OR TS="Homecare workers" OR TS="home health workers" OR TS="home health worker")

**Migration**

TS=Migration OR TS="Human Migration" OR TS=Transient* OR TS=Migrant* OR TS=Emmigrant* OR TS=Immigrant* OR TS=Emigration OR TS=Immigration OR TS=ethnic* OR TS="workforce migration"

**Migrant and Minority nurses**

TS="foreign-born nurses" OR TS="foreign-born nurse" OR TS="foreign domestic workers" OR TS="foreign domestic worker" OR TS="migrant live-in caregiver" OR TS="migrant live-in caregivers" OR TS="migrant domestic workers" OR TS="migrant domestic worker" OR TS="migrant care worker" OR TS="migrant care worker" OR TS="migrant nurse" OR TS="migrant nurses" OR TS="immigrant nurse" OR TS="immigrant nurses" OR TS="Philippine Nurses" OR TS="Philippine Nurse" OR TS="Filipino Nurse" OR TS="Filipino Nurses" OR TS="Indian Nurse" OR TS="Indian Nurses" OR TS="African Nurses" OR TS="African Nurse" OR TS="ethnic nurses" OR TS="ethnic nurse" OR TS="overseas nurse" OR TS="overseas nurses" OR TS="foreign nurse" OR TS="foreign nurses"
